# Triple synchronous primary lung cancer: a case report and review of the literature 

**DOI:** 10.1186/s13256-017-1410-4

**Published:** 2017-09-01

**Authors:** Muhammad Kashif, Puvanalingam Ayyadurai, Luong Thanha, Misbahuddin Khaja

**Affiliations:** 1 Division of Pulmonary and Critical Care Medicine, Bronx Lebanon Hospital Center, Affiliated with Icahn School of Medicine at Mount Sinai, 1650 Grand Concourse, Bronx, NY 10457 USA; 2 Department of Medicine, Bronx Lebanon Hospital Center, Affiliated with Icahn School of Medicine at Mount Sinai, 1650 Grand Concourse, Bronx, NY 10457 USA; 3 Division of Hematology and Oncology Medicine, Bronx Lebanon Hospital Center, Affiliated with Icahn School of Medicine at Mount Sinai, 1650 Grand Concourse, Bronx, NY 10457 USA

**Keywords:** Multiple primary lung cancer, Synchronous lung cancer

## Abstract

**Background:**

Multiple primary lung cancer may present in synchronous or metachronous form. Synchronous multiple primary lung cancer is defined as multiple lung lesions that develop at the same time, whereas metachronous multiple primary lung cancer describes multiple lung lesions that develop at different times, typically following treatment of the primary lung cancer. Patients with previously treated lung cancer are at risk for developing metachronous lung cancer, but with the success of computed tomography and positron emission tomography, the ability to detect both synchronous and metachronous lung cancer has increased.

**Case presentation:**

We present a case of a 63-year-old Hispanic man who came to our hospital for evaluation of chest pain, dry cough, and weight loss. He had recently been diagnosed with adenocarcinoma in the right upper lobe, with a poorly differentiated carcinoma favoring squamous cell cancer based on bronchoalveolar lavage of the right lower lobe for which treatment was started. Later, bronchoscopy incidentally revealed the patient to have an endobronchial lesion that turned out to be mixed small and large cell neuroendocrine lung cancer. Our patient had triple synchronous primary lung cancers that histologically were variant primary cancers.

**Conclusions:**

Triple synchronous primary lung cancer management continues to be a challenge. Our patient’s case suggests that multiple primary lung cancers may still occur at a greater rate than can be detected by high-resolution computed tomography.

## Background

In both men and women, lung cancer is the leading cause of cancer-related death. Risk factors associated with lung cancer include environmental risk factors, genetic factors, and tobacco smoke exposure. Patients with lung cancer are at an increased risk of developing secondary lung cancers at the same time as the first (synchronous) or later in life (metachronous). In these cases, it is critical to determine whether the secondary tumor is an independent primary tumor or a recurrence or metastasis of the first primary tumor, because this determination influences how the disease is staged and managed as well as the patient prognosis.

In 1924, the first case of two distinct primary lung cancers was published by Beyreuther *et al*. [1], and in 1975, Martini and Melamed [2] introduced the clinicopathological criteria for the diagnosis of multiple primary lung cancer (MPLC). The incidence of synchronous lung cancer (SLC) ranges from 0.2 to 8% [3], whereas continuous exposure to smoking increases the risk that a patient will develop metachronous primary lung cancer. In patients with small cell lung cancer, the overall risk of a second lung cancer is 3.5 times higher than in the general population [4]. After undergoing complete resection of non-small cell lung cancer, patients have a 1–2% risk per patient per year that of developing another lung cancer [5].

We identified a unique case of triple SLC, including adenocarcinoma, squamous cell carcinoma, and mixed small and large cell neuroendocrine carcinoma, which has poor prognostic implications. Biopsy of the other lesions helped us differentiate metastatic disease from primary lung cancer. The fact that our patient had three different primary cancers made treatment challenging.

## Case presentation

A 63-year-old homeless Hispanic man diagnosed with lung cancer 5 months previously in our hospital presented with ongoing sharp chest pain of 2 weeks’ duration, shortness of breath, chronic dry cough, and weight loss of 20 pounds within 2 months. The patient also had experienced physical activity limitations for the previous 2 months. He denied experiencing diarrhea, nausea, vomiting, or abdominal pain, and he did not have anorexia, fever, chills, or phlegm production.

The patient’s past medical history included a cerebrovascular accident with residual left-sided weakness, bronchial asthma, lung adenocarcinoma (stage IIIB) diagnosed by computed tomography (CT)-guided right upper lobe biopsy 5 months previously. He was receiving adjuvant cisplatin-based chemotherapy. His social history included 55 pack-years of smoking and a family history of cancer; his mother had colon cancer, and his brother had an unknown type of cancer. He had no environmental exposures. He was taking aspirin and atorvastatin and was using an albuterol inhaler and a fluticasone inhaler.

The patient was cachectic and afebrile on physical examination, with a blood pressure of 146/84 mmHg, and his oxygen saturation was 94% with administration of 2 L of oxygen by nasal cannula. He had bilateral grade 3 clubbing and a bronchial breath sound in the right upper lobe. Cardiovascular examination revealed normal heart sounds, and the patient’s abdomen was soft with no organomegaly. In addition, laboratory analyses of the patient’s peripheral blood and urine were normal, although his sodium levels were low.

**Fig. 1:**
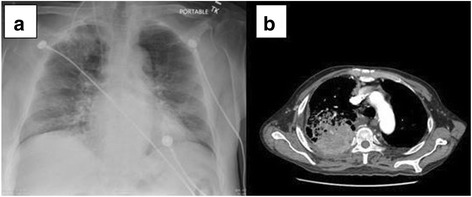
**a** Chest radiograph (anteroposterior view) showing the right portacath with an ill-defined right upper lobe mass. **b** Chest computed tomography (axial view) showing right upper lobe mass

**Fig. 2:**
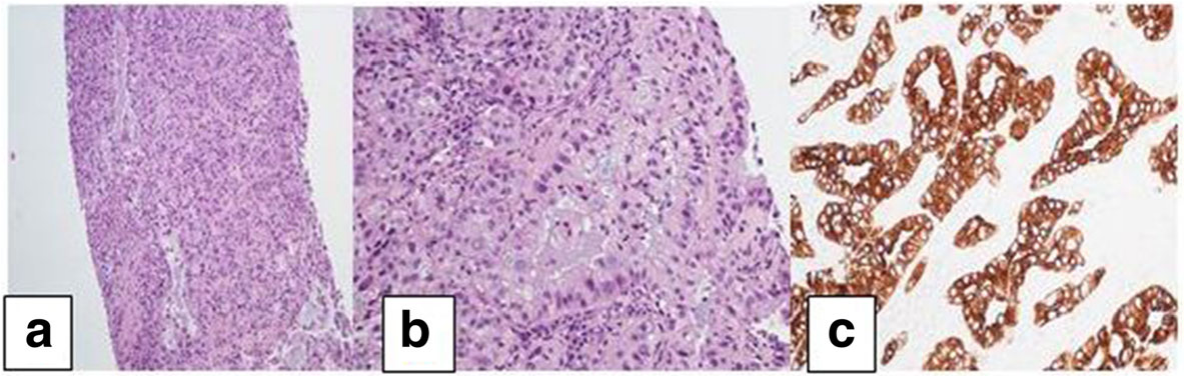
**a** Adenocarcinoma with acinar pattern shows glands with greater architectural complexity and a back-to-back arrangement (hematoxylin and eosin stain, low magnification). **b** Acinar pattern of adenocarcinoma composed of a gland with a rough oval lumen lined by malignant columnar cells with basal nuclei (hematoxylin and eosin stain, high magnification). **c** Cytokeratin 7 immunopositivity in the cytoplasm of malignant cells

**Fig. 3:**
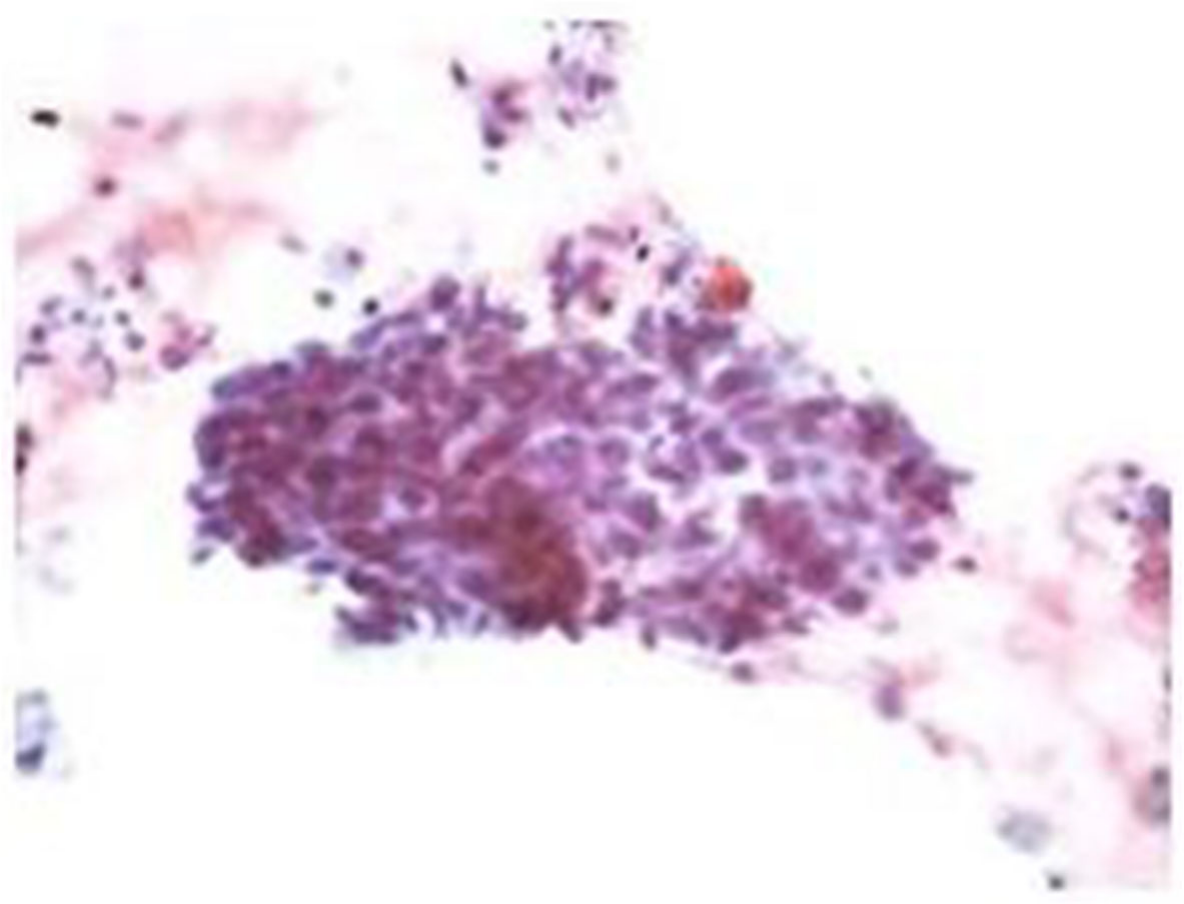
Bronchoalveolar lavage from right lung showing clusters of poorly differentiated carcinoma cells favoring the histology of squamous cell carcinoma (papanicolaou stain, high magnification)

A chest x-ray showed a persistent right upper lobe mass (Fig. 1a). This was further evaluated by review of a chest CT scan (Fig. 1b), which showed a large, rounded consolidation of the right apical parenchyma measuring 5 × 4.7 × 4 cm without necrosis or calcification. A right hilar soft tissue mass measuring 3.7 × 2.1 × 3.6 cm, consistent with focal adenopathy, was also seen. He had undergone a CT-guided lung biopsy of the mass in the right upper lobe during a previous admission, which had revealed the following immunochemical characteristics: thyroid transcription factor-1-negative, napsin-negative, cytokeratin 7 (CK7)-positive, and CK20-negative. In addition, the patient had negative test results for epidermal growth factor receptor (EGFR), K-ras, and anaplastic lymphoma kinase mutations, all consistent with adenocarcinoma (Fig. 2a–c). A bronchoscopy with bronchoalveolar lavage from the right lower lobe revealed clusters of poorly differentiated carcinoma cells, which supported the diagnosis of squamous cell carcinoma (Fig. 3). The patient was receiving cisplatin-based chemotherapy.

**Fig. 4:**
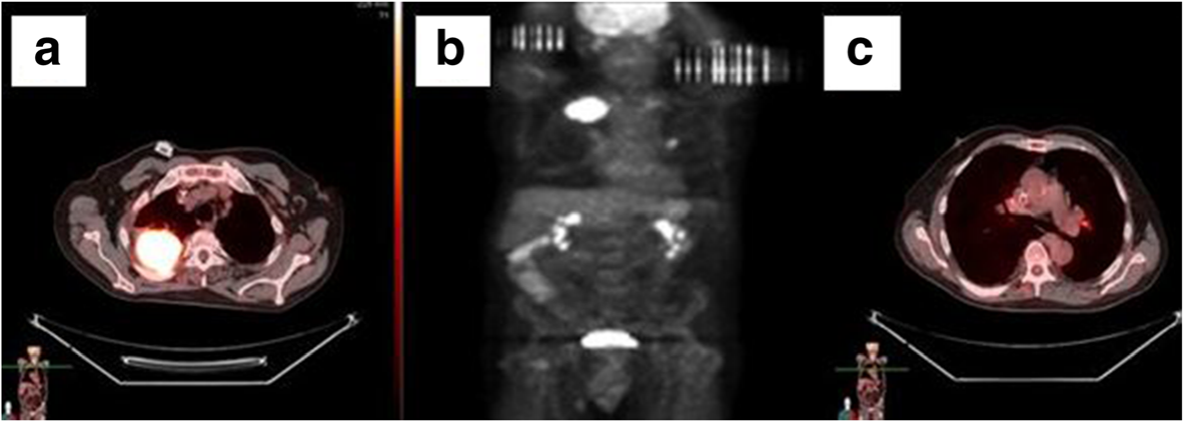
**a**, **b** Positron emission tomographic scans showing hypermetabolic activity in the right upper lobe mass. **c** Positron emission tomographic scan showing hypermetabolic activity in right and left hilar adenopathy

In view of the patient’s new symptoms of acute chest pain and shortness of breath, repeat chest CT was done, which ruled out pulmonary embolism but showed a persistent right upper lobe mass with new mediastinal lymphadenopathy. Positron emission tomography (PET) was performed, which revealed hypermetabolic activity in the 6-cm mass in the right upper lobe (standardized uptake value [SUV] 14.4), right hilar nodes (SUV 2.8), and 1.5-cm left hilar lymph nodes (SUV 4.9) (Fig. 4a–c). No hypermetabolic foci were found within the neck, abdomen, or pelvis. Results of other tests, including a brain magnetic resonance imaging scan, were negative. A pulmonary function test revealed a reduced forced expiratory volume in 1 second (FEV_1_)/forced vital capacity ratio, moderately decreased FEV_1_, and moderate obstruction.

**Fig. 5:**
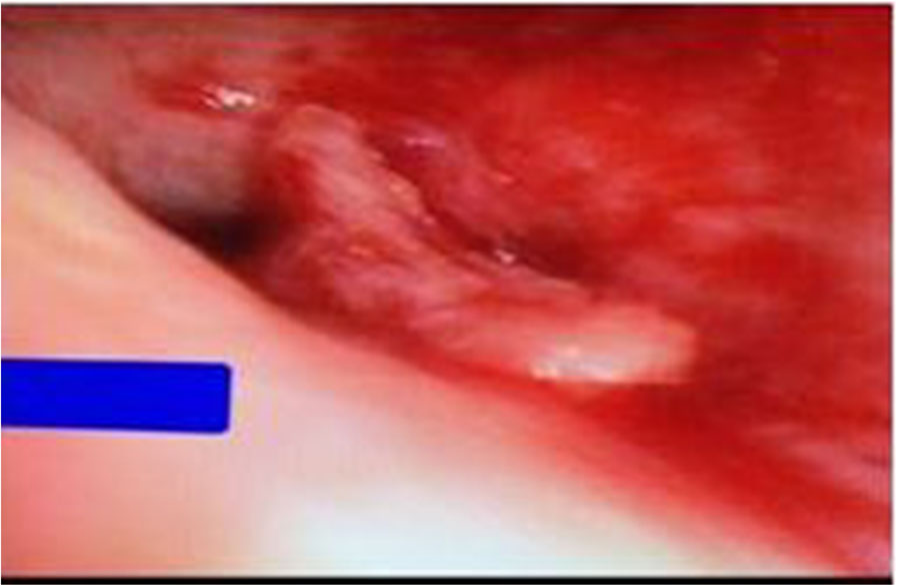
Bronchoscopic view showing an endobronchial lesion in left main stem

**Fig. 6:**
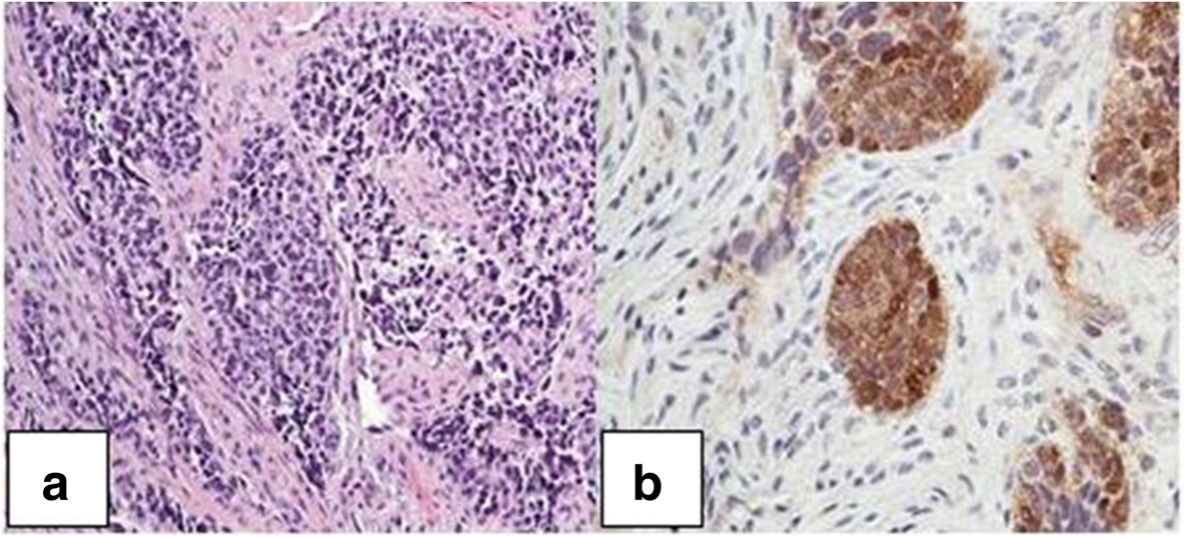
**a** Neuroendocrine carcinoma (small cell type) showing an organoid pattern (hematoxylin and eosin stain, low power). **b** High-power image showing strong immunopositivity of the neuroendocrine marker neuron-specific enolase

The patient underwent bronchoscopy for further evaluation prior to the planned mediastinoscopy. An endobronchial lesion in the left main stem (Fig. 5) was an incidental finding during the procedure. The endobronchial biopsy revealed the following immunohistochemical results: neuron-specific enolase-positive, synaptophysin-positive, CD56-positive, CK7-negative, chromogranin-negative, napsin A-negative, p63-negative, and CK5/6-negative, consistent with high-grade mixed small and large cell neuroendocrine carcinoma (Fig. 6a, b). The patient was started on chemotherapy with cisplatin and etoposide. After completing four cycles of chemotherapy, he had no significant response to the treatment. Owing to functional decline, he finally chose hospice care.

We identified a rare case of triple SLC, including adenocarcinoma, squamous cell carcinoma, and mixed small and large cell neuroendocrine carcinoma.

## Discussion

MPLC can be synchronous (occurring simultaneously) or metachronous (occurring at different times) [6]. The criterion for SLC classification, as described by Martini and Melamed *et al*. [2], is a secondary tumor that is physically distinct and separate than the primary or a secondary tumor that has a different histology from the primary tumor; if it has the same histology, it must be located in a different segment or lobe. The definition of metachronous MPLC includes having a secondary tumor that is histologically different from the primary tumor or a histologically identical tumor that occurs at least 2 tumor-free years after the primary tumor, is located in a different lobe, and arises in the absence of extrapulmonary metastases at diagnosis [3].

Antakli *et al*. [7] made modifications to the criteria of Martini and Melamed [2]. They added a further definition to the metachronous criteria for secondary tumors that have the same histology as the primary tumor. These criteria for metachronous MPLC include the presence of two or more of the following characteristics: are anatomically distinct from the primary tumor, have an associated premalignant lesion, have different deoxyribonucleic acid (DNA) ploidy, and have no systemic metastases or mediastinal spread [7].

There are multiple challenges associated with diagnosing a second primary lung cancer. First, if the secondary tumor arises within 2 years of the primary tumor, it is challenging to determine whether that tumor is a new primary tumor or a result of a residual metastasis from the original primary tumor. Second, if the secondary tumor is of the same histological type, it complicates the diagnosis of a new cancer because it is plausible that a secondary tumor of the same histological type could be an extension of the original cancer. Third, if the secondary tumor arises in an area impacted by radiotherapy (given as a treatment for the primary tumor), there can be further uncertainty whether the secondary tumor is a result of metastasis from the original tumor rather than a new cancer. Additionally, a synchronous second primary cancer judged to have associated metastatic disease would classify the patient as stage IV, which would likely contraindicate a potentially curative resection. The erroneous designation of a metachronous second primary cancer as a local recurrence might have similar therapeutic implications. A steadily growing hypothesis, known as *field cancerization*, suggests that carcinogenic exposure or genetic factors affect tissues or organs, potentiating many cells in the same area to become transformed [8].

Analysis of the clonal origin of tumors can help determine whether multiple lung tumors arise from the same clone and therefore the same tumor. Mutations in the *p53* tumor suppressor gene (chromosome 17) occur frequently in lung carcinoma, with rates up to 70% in small cell lung cancer and 50% in non-small cell lung cancer. Studies have shown that *p53* mutational analysis is good diagnostic tool for diagnosing multiple SLC in 35 to 66% of cases [9]. In addition, mutation of EGFR is a common early event in lung cancer pathogenesis, and therefore it is a useful marker, alone or with p53, for differentiating the clonal origin of lung tumors, especially when multiple tumors have similar histopathological features [10]. Analysis of loss of heterozygosity is also common. Tumors that derive from a single clone may contain cell populations that harbor similar genetic events, including loss of heterozygosity [11]. This analysis has enhanced the ability to distinguish between independent primary tumors and recurrence or metastasis [12].

Multiple synchronous primary lung cancers should be treated as two separate and distinct tumors, including their staging and treatment [13]. Management of metachronous lung cancer depends on various factors, including patient pulmonary reserve, comorbid conditions, and clinical staging of the secondary lung cancer at diagnosis. The American College of Clinical Pharmacy guidelines for treating patients with MPLC (synchronous or metachronous) recommend extrathoracic imaging (head CT/magnetic resonance imaging, and either abdominal CT with a bone scan or PET-CT) and invasive mediastinal staging before curative surgical resection [14]. Patients with synchronous or metachronous MPLC are often treated surgically if there will be sufficient pulmonary reserve after multiple lesions are resected. This is illustrated in a retrospective analysis by Battafarano *et al*., who showed that surgical intervention is safe and effective for patients with resectable metachronous lung cancer and good pulmonary reserve [15]. However, patients with limited pulmonary reserve might be limited to treatment by limited resection (e.g., segmentectomy or wedge resection), or definitive nonoperative local therapy. Patients who do not qualify for surgical resection may receive conventional radiation therapy, stereotactic whole-body radiation therapy (also called *stereotactic ablative radiotherapy*), or percutaneous image-guided tumor ablation [16, 17].

Multiple retrospective analyses have illustrated the prognostic implications of MPLC. Lee *et al*. [18] demonstrated that early tumor staging is the key factor in determining survival following surgical resection in metachronous lung cancer. Riquet *et al*. [19] demonstrated a lower overall 5-year survival rate in patients with synchronous MPLC than in patients with metachronous MPLC; however, the 5-year survival rate was increased in patients with synchronous MPLC in one lobe compared with those with synchronous MPLC in another lobe. Additionally, Rosengart *et al*. [20] reported significantly higher survival rates for patients with synchronous or metachronous MPLC than in those with metastatic or locally recurrent disease (patients with synchronous or metachronous MPLC reported to have a 23% 5-year survival rate), and Jiang *et al*. [21] illustrated that patients with MPLC had increased overall survival compared with patients with intrapulmonary metastasis. Ha *et al*. [22] showed that patients with stage I metachronous second primary lung cancer have worse survival than those who present with their first primary lung cancer.

There are four histological lung tumor types: adenocarcinoma, squamous cell, and mixed large and small cell carcinoma. Usually, lung cancers have a single histological type; however, there are reports of patients with MPLC with different histology, including our patient. Jung-Legg *et al*. [23] reported a synchronous MPLC composed of a small cell carcinoma, bronchial carcinoid, and adenocarcinoma of the right lung, and Wcisło *et al*. [24] described an MPLC composed of an adenocarcinoma, squamous cell carcinoma, and neuroendocrine carcinoma. Yoon *et al*. [25] reported a synchronous triple primary lung cancer that included a squamous cell cancer, invasive mucinous, and nonmucinous adenocarcinoma. Our patient’s case is particularly interesting and rare. In a single patient, we identified three primary lung cancers (triple SLC), classified as adenocarcinoma, squamous cell carcinoma, and mixed small and large cell neuroendocrine carcinoma.

## Conclusions

MPLC can be defined as synchronous or metachronous, and the management of patients with MPLC continues to be a challenge. Although the use of high-resolution CT has increased the ability to diagnose MPLC, our patient’s case suggests that MPLCs may still occur at a greater rate than can be detected.

## Abbreviations

CT: Computed tomography
EGFR: Epidermal growth factor receptor
FEV_1_: Forced expiratory volume in 1 second
MPLC: Multiple primary lung cancer
PET: Positron emission tomography
SLC: Synchronous lung cancer
SUV: Standardized uptake value
